# Supplementary material for: Nonintrusive thermal-wave sensor for operando quantification of degradation in commercial batteries
Source: Nat Commun. 2023 Dec 11;14:8203. doi: 10.1038/s41467-023-43808-9 (PMC10713567; doi:10.1038/s41467-023-43808-9)
Supplement: Supplementary file 1 — Supplementary Information [file 41467_2023_43808_MOESM1_ESM.pdf]

Nonintrusive thermal-wave sensor for operando quantification of degradation in commercial batteries

Yuqiang Zeng[1,2], Fengyu Shen[2], Buyi Zhang[2,3], Jaeheon Lee[2,4], Divya Chalise[2,3], Qiye Zheng[2,5], Yanbao Fu[2], Sumanjeet Kaur[2], Sean D. Lubner[2], Vincent S. Battaglia[2], Bryan D. McCloskey[2,4], Michael C. Tucker[2], Ravi S. Prasher[2,3*]

[1]School of Microelectronics, Southern University of Science and Technology, Shenzhen, 518055, China

[2]Energy Storage and Distributed Resources Division, Lawrence Berkeley National Laboratory, Berkeley, CA, 94720, USA

[3]Department of Mechanical Engineering, University of California, Berkeley, CA, 94720, USA

[4]Department of Chemical and Biomolecular Engineering, University of California, Berkeley, CA, 94720, USA

[5]Mechanical and Aerospace Engineering Department, The Hong Kong University of Science and Technology, Hong Kong, China

[*]Corresponding author: rsprasher@lb.gov

Supplementary Table 1. Materials & Properties

Graphite anode	Cu foil thickness: 10 µm<br>Coating thickness: 70 µm<br>Bulk porosity: 37.4%<br>Coating loading: 9.38 mg/cm2<br>Coating density: 1.34 g/cm3<br>Estimated areal capacity: 2.84 mAh/cm2<br>91.83 wt% Superior Graphite SLC 1506T<br>2 wt% Timcal C45 carbon<br>6 wt% Kureha 9300 PVDF binder<br>0.17 wt% Oxalic acid
NMC cathode	Al foil thickness: 20 µm<br>Coating thickness: 71 µm<br>Bulk porosity: 33.1%<br>Coating loading: 18.57 mg/cm2<br>Coating density: 2.62 g/cm3<br>Estimated areal capacity: 2.67 mAh/cm2<br>90 wt% Toda NMC532<br>5 wt% Timcal C45 carbon<br>5 wt% Solvay 5130 PVDF binder
Separator (Celgard 2400)	Thickness: 25 µm<br>Bulk porosity: 41%
Gen 2 Electrolyte	1.2 M LiPF6 in EC:EMC (3:7)

Supplementary Table 2. Thermophysical properties of a unit cell.

	Cu CC	Graphite anode	Anode particle	NMC cathode	Cathode particle	Separator	Separator solid part	Al CC
Dimension* (μm)	10	70	~4	71	~4	25	-	20
Bulk porosity	-	37.4%	-	33.1%	-	41%	-	
k_{wet}# (W/mK)	401	1.30	2.82	1.03	1.95	0.30	0.35	237
k_{dry}+ (W/mK)	Same as wet	1.06	Same as wet	0.82	Same as wet	0.17	Same as wet	Same as wet
TCR_{wet}# (m^2K/W)	2.79×10^{-4}							
TCR_{dry}+ (m^2K/W)	6.04×10^{-4}							

*: the layer thickness or particle radius.

#: wet for the fully wet condition by Gen 2 electrolyte (k_f = 0.23 W/mK).

+: dry for the fully dry condition with gas (k_f = 0.025 W/mK).

Supplementary Table 3. Estimation of ϕ_{dry} from the measured mass difference

	EMC evaporation (g)	EMC consumption (g)	EMC consumption (vol%)	ϕ_{dry} from mass difference (%)	ϕ_{dry} from our method (%)
Pristine cell	4.5927	-	-	-	-
Aged cell ($\bar{T}_{ch} = 25$ ℃)	1.8312	2.7615	45.28	60.13±12.35	62.85
Aged cell ($\bar{T}_{ch} = 40$ ℃)	1.9827	2.6100	42.80	56.83±12.35	58.20

Supplementary Table 4. Quantity of Li-containing species in pristine and aged graphite anodes

	Dead Li and lithiated graphite Li_xC_6 (μmol/cm^2)	Solid carbonate species LiEMC, Li_2CO_3 (μmol/cm^2)	Lithium acetylide Li_2C_2 (μmol/cm^2)
Pristine Anode	0.6523	0.2988	0.0253
Aged Anode ($\bar{T}_{ch}$= 25 °C)	2.1413	0.5908	0.0459
Aged Anode ($\bar{T}_{ch}$= 40 °C)	0.7000	0.3855	0.03685

Supplementary Table 5. Thermal resistance of unit cells and case in various battery formats

	Case		Unit cells (electrodes, separators, and current collectors)		R_{case} (m^{2}K/W)	$R_{uc,tot}$ (m^{2}K/W)	$R_{uc,tot}$ $/R_{case}$
	Thickness (mm)	k_{case} (W/m-K)	Thickness (mm)	k_{uc} (W/m-K)			
Pouch cells 102.5×51×6 mm^3	0.11	0.39	5.78	0.40	2.80×10^{-4}	0.0145	51.6
Cylindrical cells (18650)	0.25	45	7.75		5.63×10^{-6}	0.0488	8662.1
Prismatic cells 173×125×45 mm^3	1.1	237	42.8		4.64×10^{-6}	0.107	23053.6

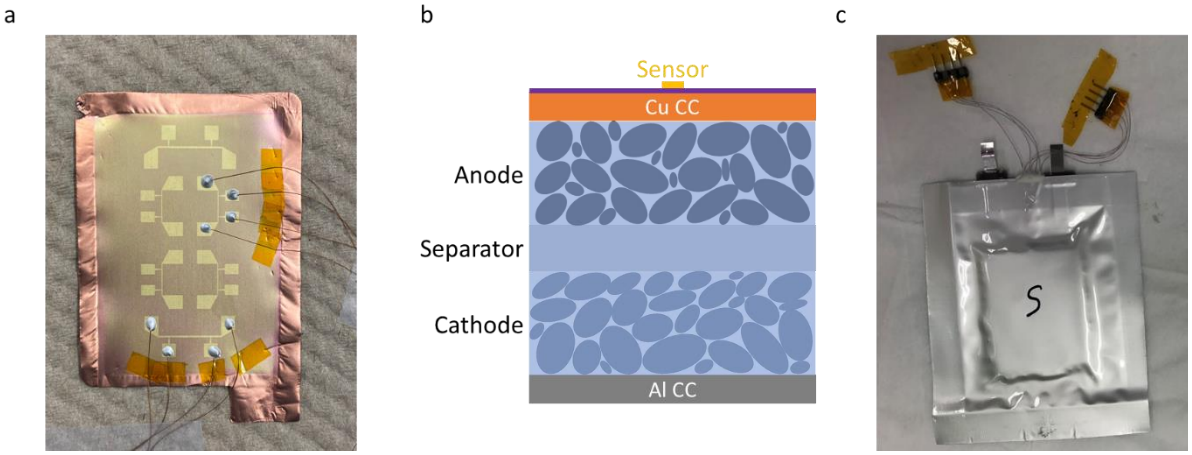

Supplementary Figure 1 | Thermal wave sensor embedded batteries of single unit cells for calibration and validation. a, thermal wave sensor on the Cu current collector with an electrical insulation layer in between. b, schematics of the sensor for measuring the thermal conductivity of a unit cell. c, pouch cells with embedded thermal wave sensors.

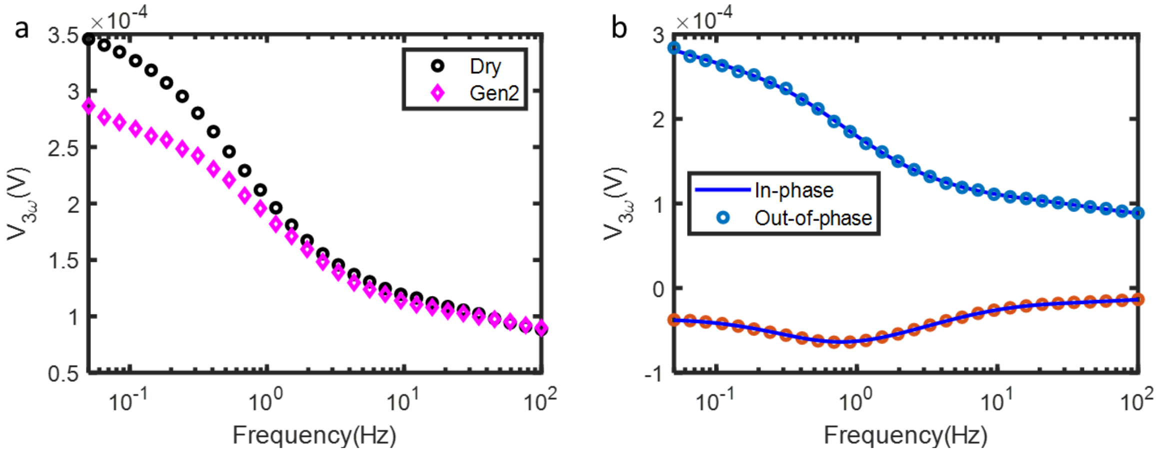

Supplementary Figure 2 | Calibration experiments by measuring the k_{eff} in fully dry and wet conditions. a, raw data collected in completely dry and wet conditions. b, representative fit to extract the k_{eff} for the cell wetted by Gen 2 electrolyte.

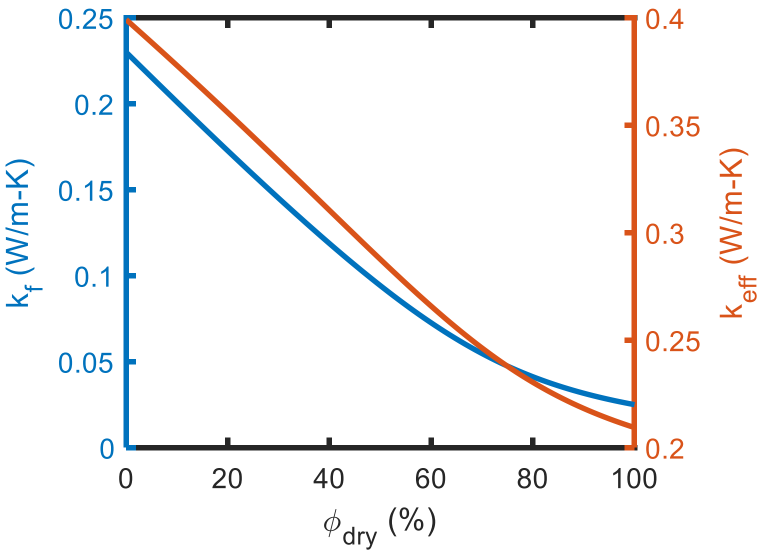

Supplementary Figure 3 | The impact of electrolyte consumption on the effective k_f and measured k_{eff}. Based on this relationship, the amount of electrolyte consumption can be back calculated from the measured k_{eff} and extracted k_f.

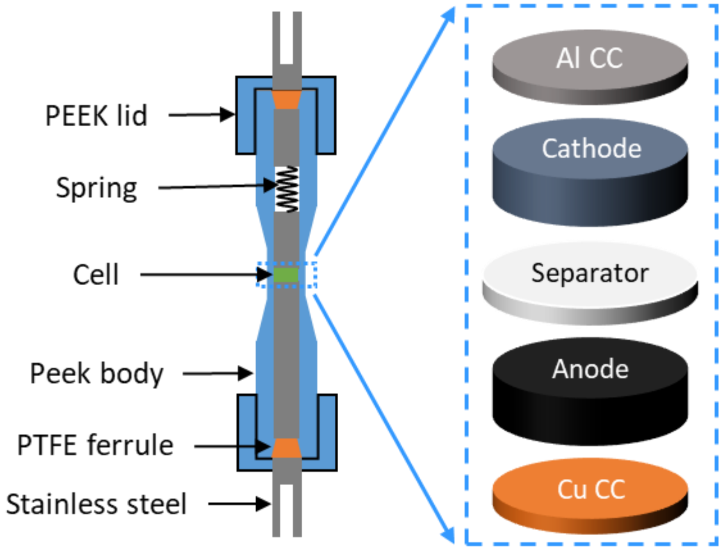

Supplementary Figure 4 | Schematics of our custom PEEK cell holder and the components inside the cell. The main body of the cell holder was machined from polyether ether ketone (PEEK) for the transmission of X-ray. PTFE ferrule was used to keep the cell airtight. The contact between the cell and stainless-steel pins was adjusted using a hard spring.

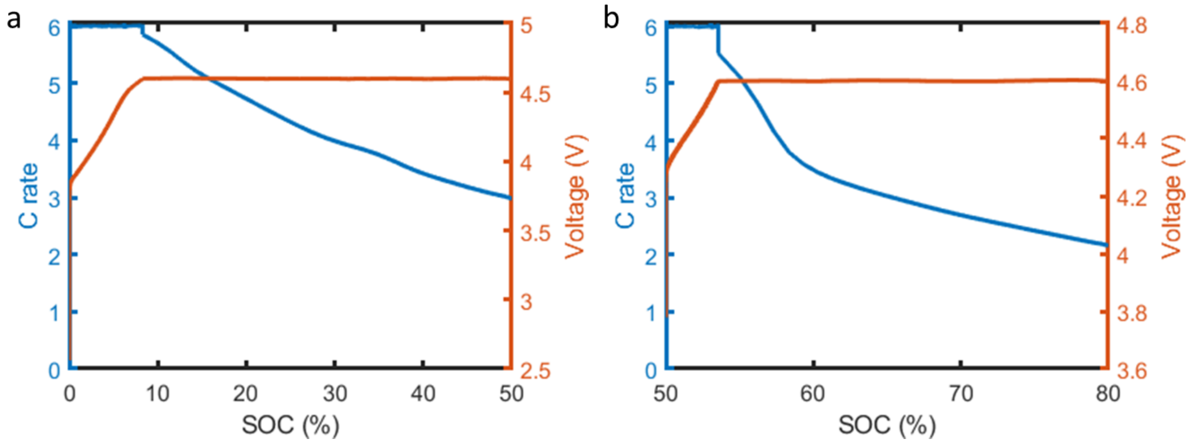

Supplementary Figure 5 | Evolution of C rate and voltage during 6C CCCV charging to a) 50% SOC and b) 80% SOC. A high cutoff voltage of 4.6 V was used to maintain the high C rate during charging and cause sufficient Li plating for the observation purpose. The microtomography was conducted right after charging to the set SOC.

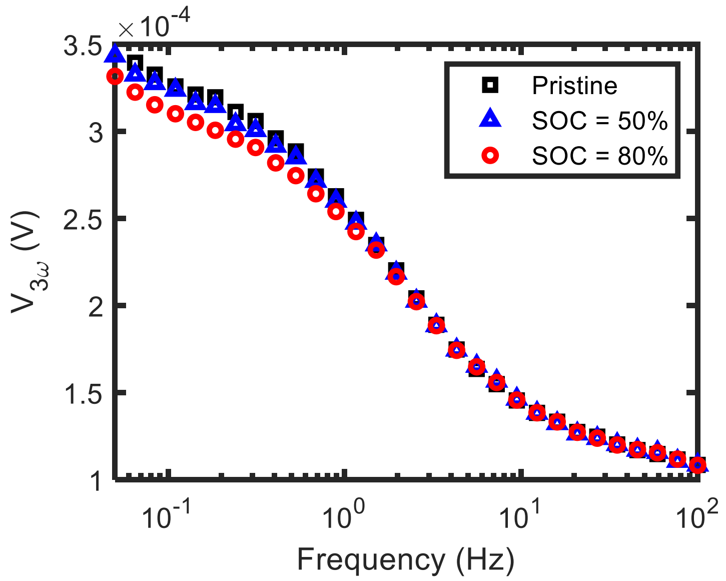

Supplementary Figure 6 | Raw data collected for the cells at SOC = 0, 50% and 80%. Lithium plating causes a decrease of $V_{3\omega}$, which indicates the rise of k_{eff}.

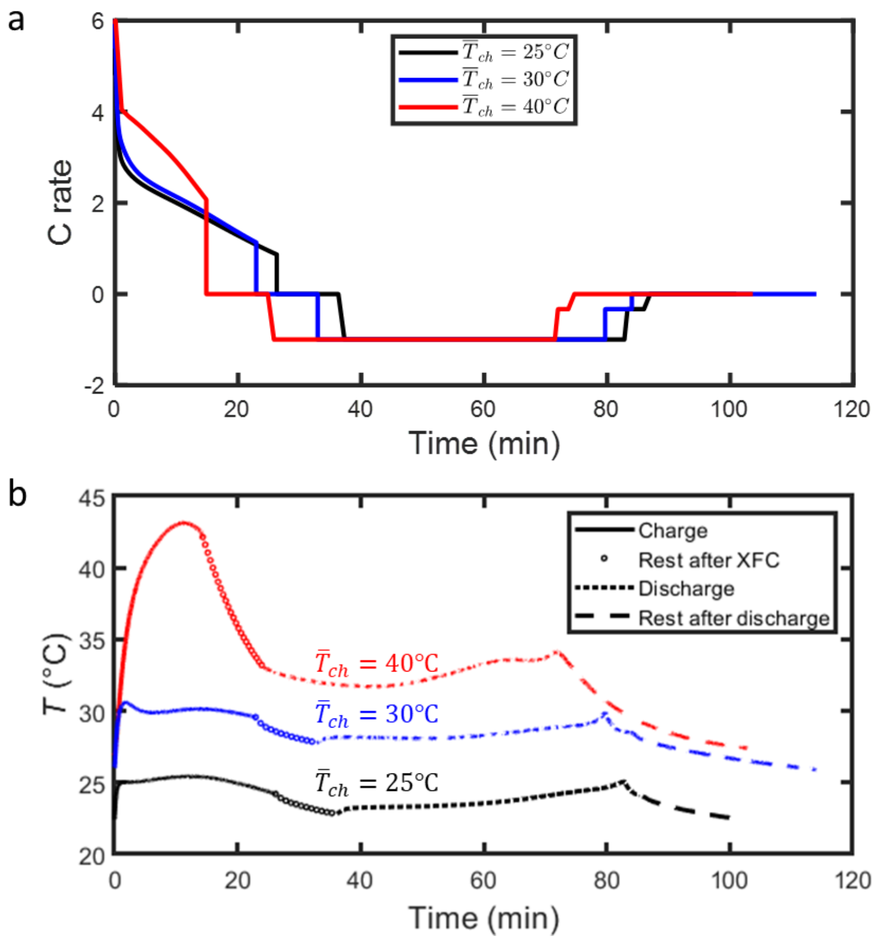

Supplementary Figure 7 | Representative evolution of a) C rate and b) temperature in a fast-charging cycle for the cells under different thermal conditions. The effective C rate increases with the charging temperature. The rest time after discharge varies from 15 to 30 min for restoring the approximate thermal equilibrium in different cases.

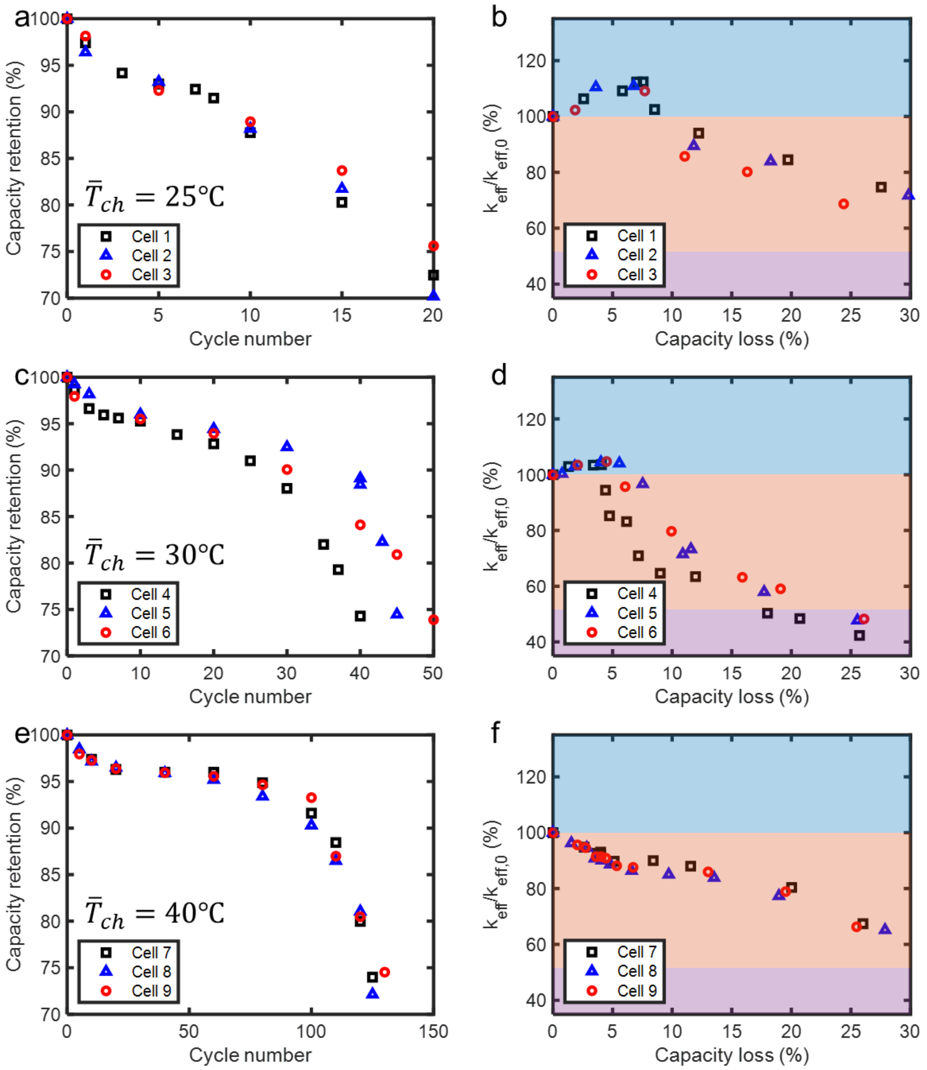

Supplementary Figure 8 | Summary of battery degradation analysis for 9 cells. Capacity retention of the cells with a) $\overline{T}_{ch}$ = 25 °C, c) $\overline{T}_{ch}$ = 30 °C, and e) $\overline{T}_{ch}$ = 40 °C. Evolution of k_{eff} during cycling for the cells under different thermal conditions, *i.e.*, b) $\overline{T}_{ch}$ = 25 °C, d) $\overline{T}_{ch}$ = 30 °C, and f) $\overline{T}_{ch}$ = 40 °C. 3 cells were tested for each thermal condition for ensuring the measurement repeatability. Although the cell performance differs slightly due to the cell-to-cell variation, the cells tested in the same thermal condition degrade in a similar manner. The results of cells 1, 4, and 7 were presented and discussed in the manuscript.

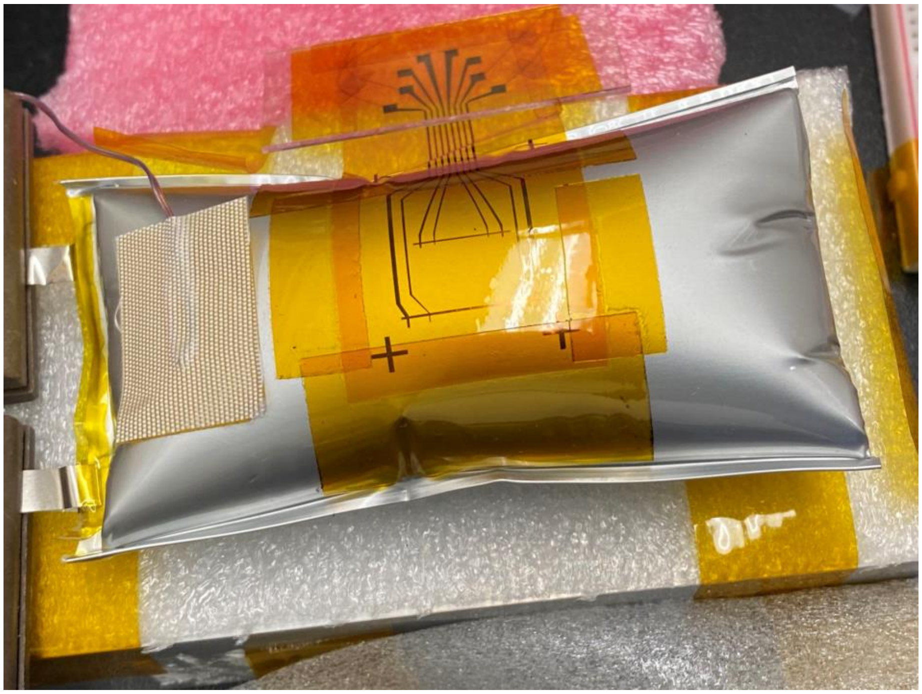

Supplementary Figure 9 | Cell swelling observed in the case of $\overline{T}_{ch}$ = 30 °C.

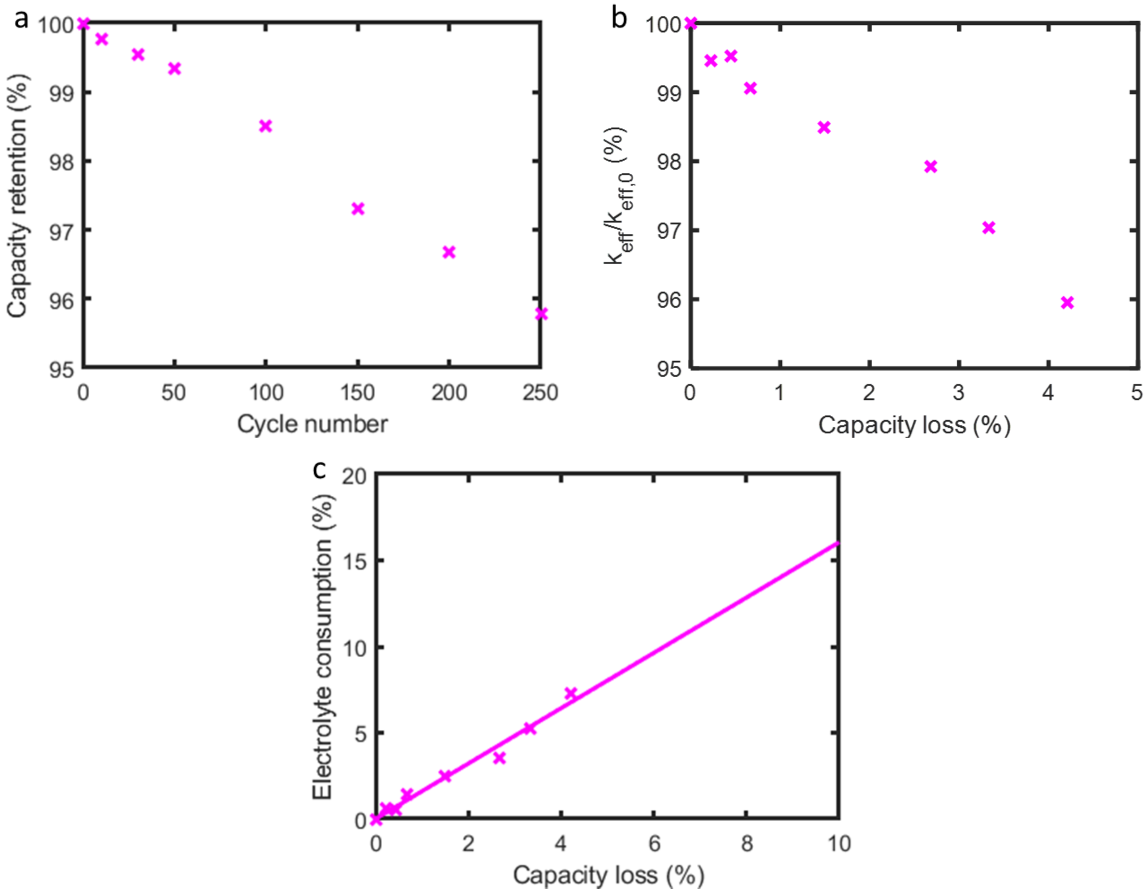

Supplementary Figure 10 | Calibration of the electrolyte consumption rate due to SEI growth on graphite during 1C1C cycling. a, Evolution of capacity retention with cycle number. The linear capacity fade is attributed to the dominant degradation mechanism during slow 1C charging, *i.e.*, the growth of SEI on graphite. b, Decrease of k_{eff} with capacity loss due to electrolyte consumption. c, Calibrated electrolyte consumption *vs.* capacity loss. The electrolyte consumption rate associated with SEI growth on graphite is nearly constant based on the linear increase of electrolyte consumption with capacity loss. Note that we collected the data within 5% capacity loss where aging induced Li plating unlike happens.

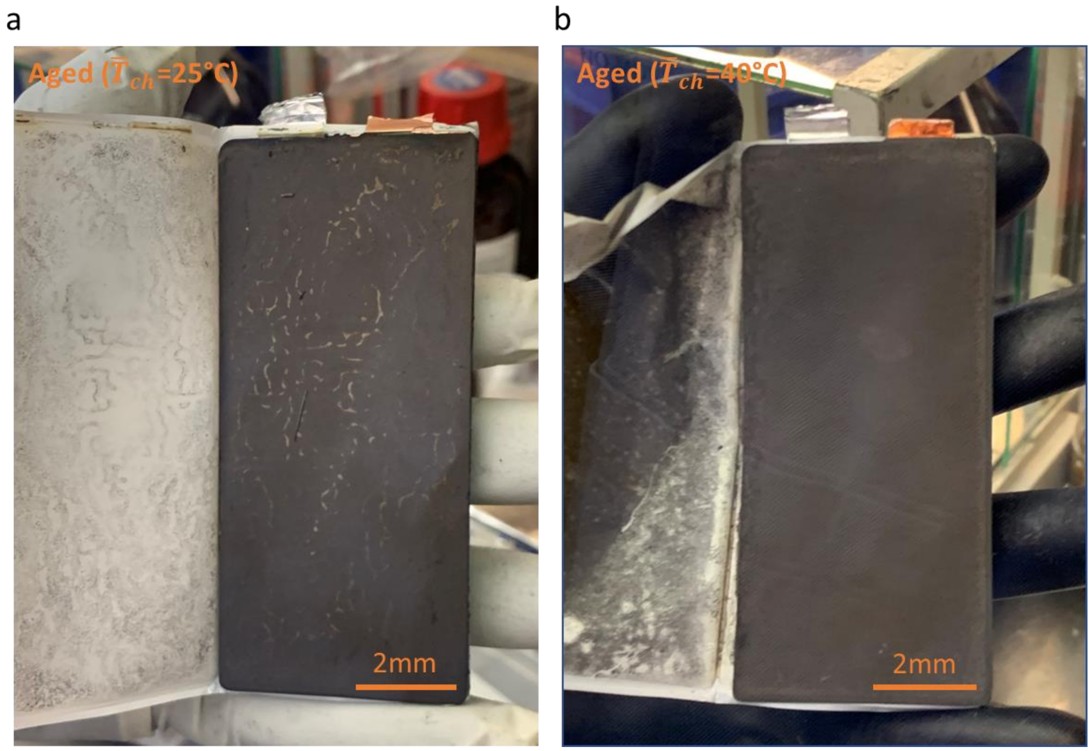

Supplementary Figure 11 | Images of aged graphite anode from the case of a) $\bar{T}_{ch}$ = 25 °C and b) $\bar{T}_{ch}$ = 40 °C. Severe lithium plating appears only in the low $\bar{T}_{ch}$ case. The images were taken in a glove box and representative pieces were sealed in a sample holder for optical characterization using a confocal microscope.

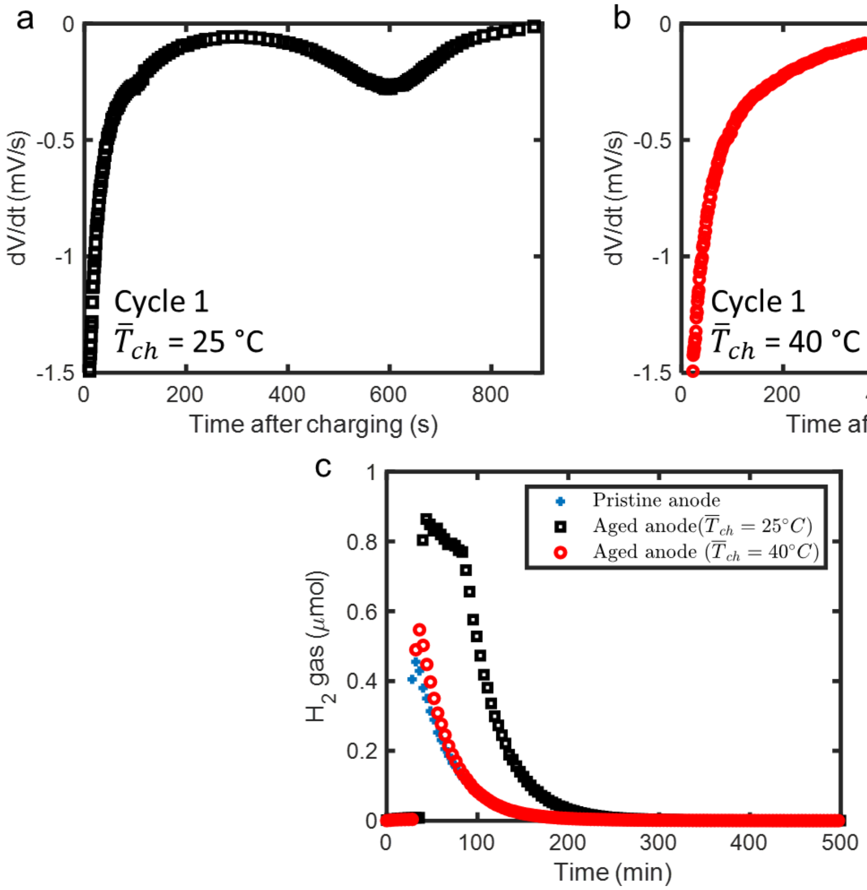

Supplementary Figure 12 | Validation of mitigated lithium plating at $\overline{T}_{ch}$ = 40 °C. The derivative of resting voltage after first-cycle fast charging with respect to time for a) $\overline{T}_{ch}$ = 25 °C and b) $\overline{T}_{ch}$ = 40 °C. The inflection point feature observed in the case of $\overline{T}_{ch}$ = 25 °C confirms the severe lithium plating, while this feature disappears for $\overline{T}_{ch}$ = 40 °C. c, H_2 gas evolution during titration for pristine and aged graphite anodes. For the cells discharged to 2.75V, the quantity of dead lithium and lithiated graphite in the anode can be determined from the accumulated amount of H_2 gas (Supplementary Table 4). The quantitative analysis verifies the mitigation of lithium plating during fast charging by operating at higher temperatures.

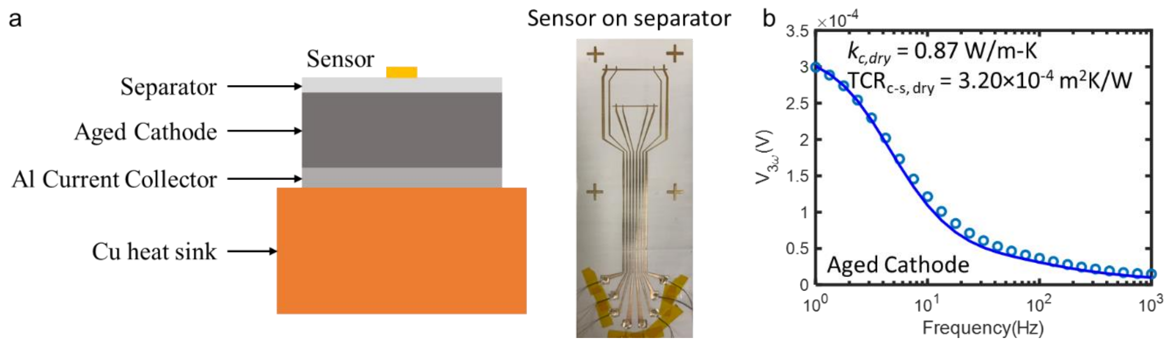

Supplementary Figure 13 | Variation of thermal transport properties due to cathode aging. a, schematics of thermal measurement for the aged cathode, with a sensor fabricated on the separator. b, extraction of the thermal conductivity and thermal contact resistance by fitting to the raw data. The measured cathode thermal conductivity ($k_{c,\,dry}$) and thermal contact resistance ($TCR_{c\text{-}s,\,dry}$) in a dry condition is 0.87 W/m-K and 3.20×10^{-4} m^2K/W, respectively. From our case studies, the relative variation of k_{eff} associated with cathode change is only 0.57%, which is within the uncertainty range of our non-embedded measurement and negligible compared to the effect of lithium plating and electrolyte consumption.

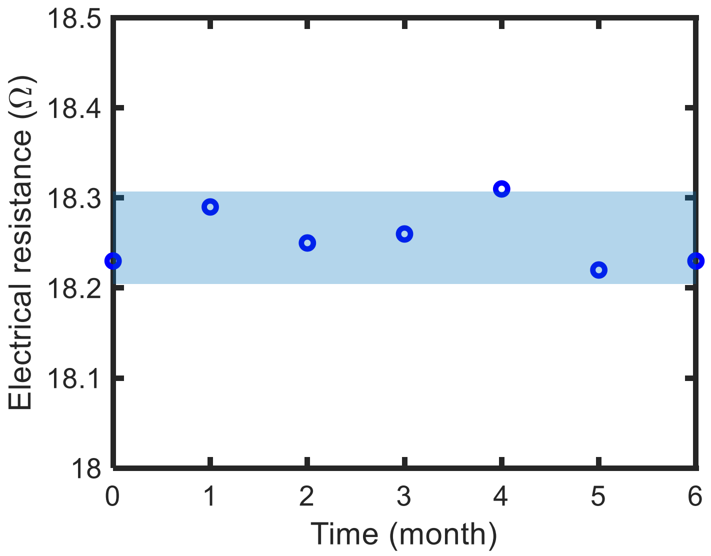

Supplementary Figure 14 | Sensor stability in 6 months. The variation of electrical resistance of our sensor is comparable to that associated with the temperature fluctuation of the temperature chamber (±1 °C; shaded area in the plot), which verifies the sensor stability in the long term.

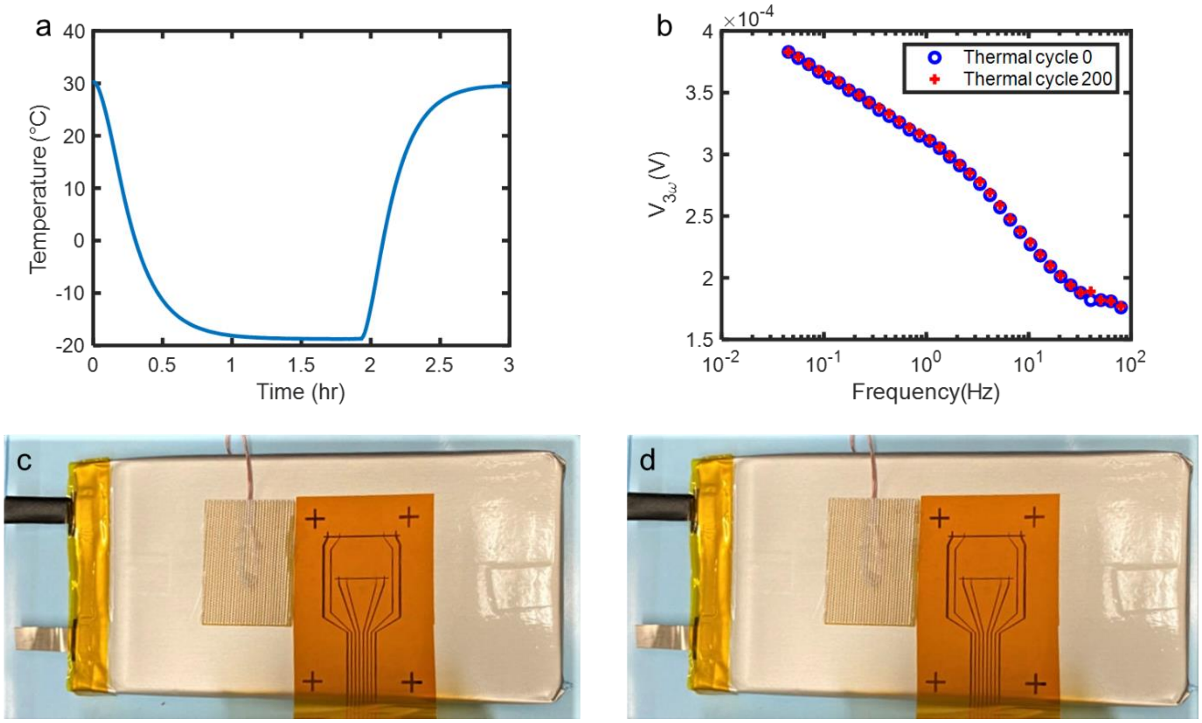

Supplementary Figure 15 | Sensor bonding reliability across a wide range of temperatures. a, temperature profile of a thermal cycle between 30 °C and -20 °C. b, thermal-wave signals collected before and after 200 thermal cycles. Optical images of the sensor c) before and d) after thermal cycling. No degradation of bonding is observed from these characterizations.

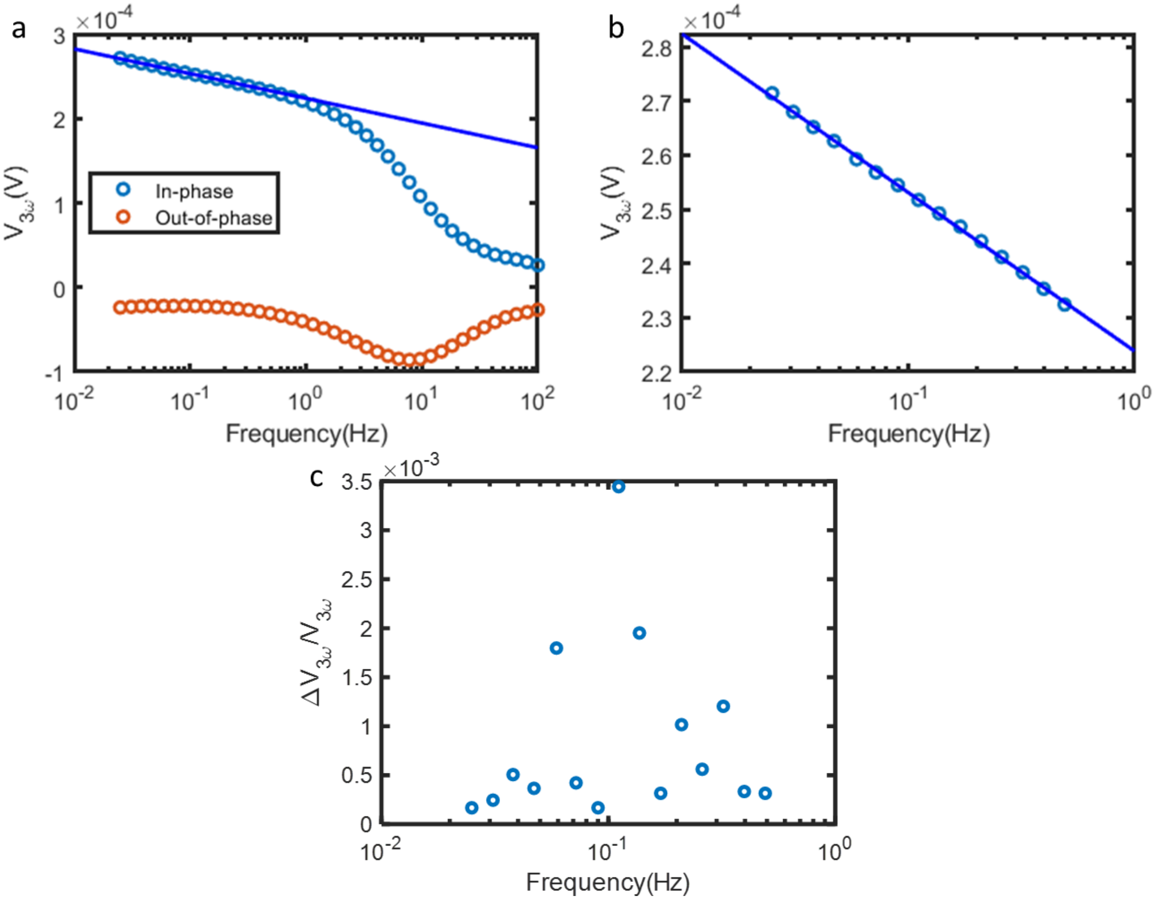

Supplementary Figure 16 | Low-frequency thermal signals for the k_{eff} measurement. a, representative raw $V_{3\omega}$ data from 25 mHz to 100 Hz. As the penetration depth is inversely proportional to $\sqrt{\omega}$, the bulk thermal conductivity is sensitive to the low-frequency signals. The well-developed low-frequency slope method is used to extract the k_{eff} from the thermal signals in the range of 25mHz to 0.5Hz, and a representative fit to $V_{3\omega}$ vs. ω is shown in b). c, relative standard deviation of $V_{3\omega}$. Fitting to the $V_{3\omega}$ and $\Delta V_{3\omega}$ determines the uncertainty of k_{eff} (±0.75%).

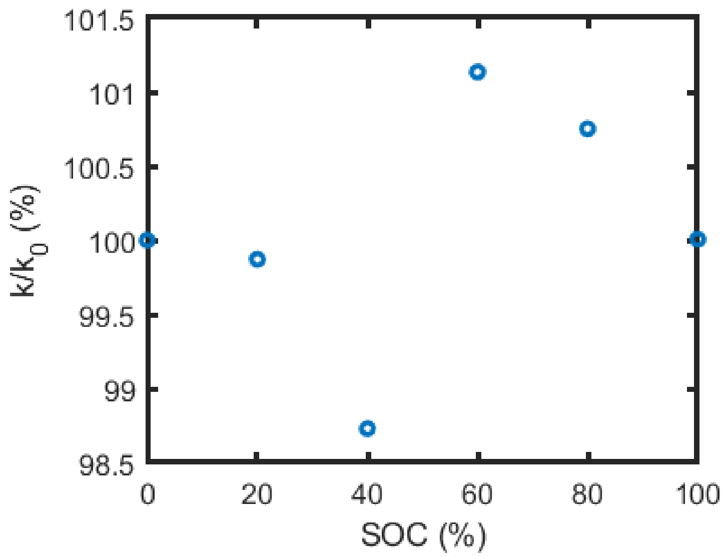

Supplementary Figure 17 | Variation of k with SOC. The dependence of k on SOC is weak and the normalized change is less than 1.5%.
