# Supplementary material for: Nonintrusive thermal-wave sensor for operando quantification of degradation in commercial batteries
Source: Nat Commun. 2023 Dec 11;14:8203. doi: 10.1038/s41467-023-43808-9 (PMC10713567; doi:10.1038/s41467-023-43808-9)
Supplement: Supplementary file 3 — Description of Additional Supplementary Files [file 41467_2023_43808_MOESM3_ESM.docx]

**Description of Additional Supplementary Files**

**File Name: Supplementary Code 1
Description:** Computational code for the thermal model: calibration, validation, and proof-of-concept study.
