# Supplementary material for: Nonintrusive thermal-wave sensor for operando quantification of degradation in commercial batteries
Source: Nat Commun. 2023 Dec 11;14:8203. doi: 10.1038/s41467-023-43808-9 (PMC10713567; doi:10.1038/s41467-023-43808-9)
Supplement: Supplementary file 4 — Supplementary Code [file 41467_2023_43808_MOESM4_ESM.zip › polyfitZero-1.3/html/polyfitZero_example.html]

polyfitZero example 

# polyfitZero example

## Contents

- LaTex
- initialize workspace
- create some data with noise
- fit data
- scale data
- get error estimates
- annotate

## LaTex

## initialize workspace

```
close('all'),clear('all'),clc
```

## create some data with noise

```
x = 1:10;y = (x+rand(1,10)/10).^2;
```

## fit data

```
degree = 2;
p = polyfitZero(x,y,degree);
for n = 1:degree,fprintf('p%d = %f\n',n,p(n)),end
```

```
p1 = 0.995270
p2 = 0.123069
```

## scale data

```
[p,~,mu] = polyfitZero(x,y,degree);
fprintf('\nScale X:\n')
for n = 1:degree,fprintf('p%d = %f\n',n,p(n)),end
fprintf('scaled by %f\n',mu(2))
```

```
Scale X:
p1 = 9.123306
p2 = 0.372611
scaled by 3.027650
```

## get error estimates

```
[p,S,mu] = polyfitZero(x,y,degree);
[yest,derr] = polyval(p,x,S,mu); % fit to data, calculate error
plot(x,y,'o'),hold('all'),grid
errorbar(x,yest,derr),title('Polynomial fit forcing y through origin.')
xlabel('x'),ylabel('y'),legend('data','fit','Location','NorthWest')
```

## annotate

```
pos = get(gca,'Position');
xl = 11;xlim([0,xl]),yl=120;ylim([0,yl])
for n = 1:numel(x)
    xpos = pos(1)+pos(3)*x(n)/xl;ypos = pos(2)+pos(4)*yest(n)/yl;xtrim = -0.05;
    annotation('textbox',[xpos+xtrim,ypos,0.1,0.1], ...
        'LineStyle','none','FontWeight','bold', ...
        'String',sprintf('%4.2f%%',derr(n)/yest(n)*100))
end
```

Published with MATLAB® R2013a
