# Supplementary figures and images for: Nonintrusive thermal-wave sensor for operando quantification of degradation in commercial batteries
Source: Nat Commun. 2023 Dec 11;14:8203. doi: 10.1038/s41467-023-43808-9 (PMC10713567; doi:10.1038/s41467-023-43808-9)

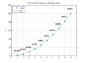

Supplement: Supplementary file 4 — Supplementary Code [file 41467_2023_43808_MOESM4_ESM.zip › polyfitZero-1.3/html/polyfitZero_example.png]

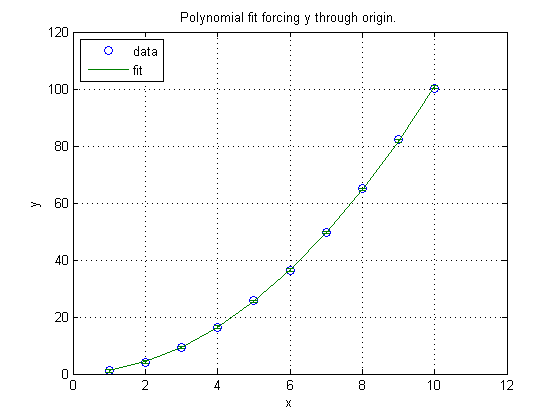

Supplement: Supplementary file 4 — Supplementary Code [file 41467_2023_43808_MOESM4_ESM.zip › polyfitZero-1.3/html/polyfitZero_example_01.png]

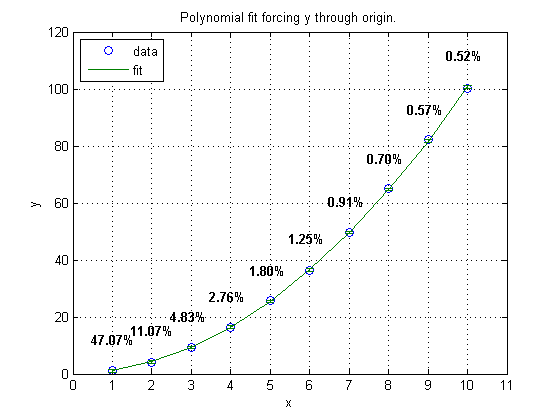

Supplement: Supplementary file 4 — Supplementary Code [file 41467_2023_43808_MOESM4_ESM.zip › polyfitZero-1.3/html/polyfitZero_example_02.png]

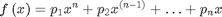

Supplement: Supplementary file 4 — Supplementary Code [file 41467_2023_43808_MOESM4_ESM.zip › polyfitZero-1.3/html/polyfitZero_example_eq30592.png]

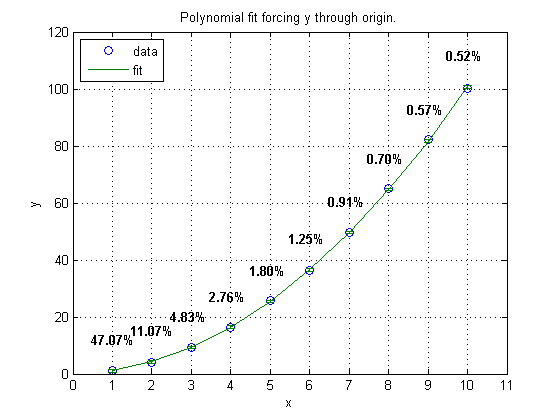

Supplement: Supplementary file 4 — Supplementary Code [file 41467_2023_43808_MOESM4_ESM.zip › polyfitZero-1.3/polyfitZero_example_plot.png]
